# Quantification of Hantaan Virus with a SYBR Green Ⅰ-Based One-Step qRT-PCR Assay

**DOI:** 10.1371/journal.pone.0081525

**Published:** 2013-11-21

**Authors:** Wei Jiang, Hai-tao Yu, Ke Zhao, Ye Zhang, Hong Du, Ping-zhong Wang, Xue-fan Bai

**Affiliations:** Center of infectious diseases, Tangdu Hospital, Fourth Military Medical University, Xi’an, Shaanxi, China; University of Texas Medical Branch, United States of America

## Abstract

Hantaan virus (HTNV) is a major zoonotic pathogen that causes hemorrhagic fever with renal syndrome (HFRS) in Asia, especially in China. Shaanxi province, which is located in northwest of China, is one of the areas in China most severely afflicted with HFRS epidemics annually. This study aims to establish a quantitative RT-PCR (qRT-PCR) assay to detect HTNV both in cell culture and clinical serum samples. We established a SYBR Green Ⅰ-based one-step qRT-PCR assay that targets the S segment of the HTNV genome for rapid detection and quantification. The HTNV cRNA standards were constructed by *in vitro* transcription, and the copy numbers of the HTNV cRNA were quantified. Standard curve was generated by determining the mean cycle threshold (Ct) values versus 10-fold serial dilutions of the HTNV cRNA over a range of 1×10^8^ to 1×10^3^ copies/μl. The standard curve had a reaction efficiency of 102.1%, a correlation coefficient (R^2^) of 0.998, and a slope of -3.273. The coefficient of variation (CV) of the intra- and inter-assays ranged from 0.68% to 3.00% and from 0.86% to 3.21%, respectively. The cycle intervals of the qRT-PCR assay between each dilution ranged from 2.9 to 3.8 cycles, and the lowest detection limit of the qRT-PCR assay was 10 copies/μl. The assay exhibited high specificity that was confirmed by melting curve analysis, and no cross reaction with the Seoul virus (SEOV) and other viruses (HBV, HCV and HIV) was observed. HTNV RNA was also detected in the 27 serum samples of clinical HFRS patients using the assay, and the HTNV RNA viral load ranged from 2.06×10^1^ to 1.95×10^5^ copies/μl. The SYBR Green Ⅰ-based one-step qRT-PCR assay is a sensitive, specific, reproducible, and simple method for detecting and quantifying HTNV in cell culture and clinical samples.

## Introduction

Hantaan virus (HTNV) is a worldwide pathogen that causes serious infectious disease featured with febrile, mucocutaneous hemorrhage, renal damage and shock. The disease is thus named hemorrhagic fever with renal syndrome (HFRS). The cases of HFRS in China account for nearly 90% of all HFRS cases worldwide [1]. During 1950-2007, 1,557,622 HFRS cases and 46,427 deaths from HFRS (a death rate of 3%) were reported in China [2], and HFRS cases are reported in most provinces and cities (28/31) of mainland China annually [2,3], making HFRS a notable public health problem. Shaanxi province, which is located in northwest of China, is one of the most seriously afflicted areas [4]. Over the past decade (2001-2010), the majority of HFRS cases (65%) occurred from October to December annually [4]. The composition of the population of HFRS cases has changed with the rapid development of society and the environment in recent years; however, the infected population is primarily 16- to 59-year-old farmers [4]. 

HTNV (genus *Hantavirus*, family *Bunyaviridae*) is an enveloped virus with a single-stranded, negative-sense RNA genome. HTNV was the first hantavirus described and was isolated in 1978 [5]. The virion encloses three gene segments named L (large), M (medium), and S (small), which encode different proteins [6]. The L segment encodes an RNA-dependent RNA polymerase, the M segment encodes two glycoproteins, Gn and Gc, and the S segment encodes a nucleocapsid protein (NP) [7]. Variation in the S and M segments may alter the virulence and antigenicity of hantaviruses [8]. The transmission pathway of hantavirus within rodents and from rodents to humans includes inhalation of aerosolized excreta (i.e., saliva, urine and feces), the consumption of contaminated food, or rodent bites [9]. Seven species of hantaviruses have been identified in China, but only HTNV, which is carried by *Apodemus agrarius*, and Seoul virus (SEOV), which is carried by *Rattus norvegicus*, are associated with HFRS [10–12]. However, the disease caused by HTNV is more clinically severe than that caused by SEOV [3]. HTNV has been reported to be the predominant etiological agent for HFRS in the Xi’an district of Shaanxi province, and SEOV and other hantavirus species are seldom detected [4]. 

In clinical practice, the commonly used diagnostic methods for detecting of HTNV are primarily based on serological techniques, such as the enzyme-linked immunosorbent assay (ELISA) and immunofluorescence assay (IFA). Other molecular biological methods, such as conventional RT-PCR and nested PCR, have also been developed but are mainly used for experimental research. Because it is time-consuming (usually requiring 5-7 days to perform the test), a plaque assay, which is the classical virus titration approach, is seldom used in laboratories. However, none of these detection methods provides information about the load of the infectious virions. 

With the development of the quantitative RT-PCR (qRT-PCR) assay, an easier method to quantify viral load has become available, especially for the quantifying of HBV, HCV and HIV in patient peripheral blood [13–15]. The TaqMan-based real-time PCR assay has been described for the detection and quantification of hantavirus [16–18]. 

SYBR Green Ⅰ-based qRT-PCR is technically simpler and less expensive than the TaqMan probe or molecular beacon-based assays. This method uses fluorescent dye that directly binds to the amplified double-stranded DNA sequences; therefore, the fluorescence signal is proportional to the amount of PCR products accumulated in the reaction tubes. When coupled with melting curve analysis of the amplification products, SYBR Green Ⅰ-based qRT-PCR assay offers an alternative choice to TaqMan-based assays [15]. 

In this study, a SYBR Green Ⅰ-based one-step qRT-PCR assay was established to target the S segment of the HTNV genome for quantification of the HTNV RNA viral load, and the performance of the qRT-PCR assay was evaluated using serum samples from HFRS patients.

## Results

### Synthesis of the HTNV cRNA

**Figure 1:**
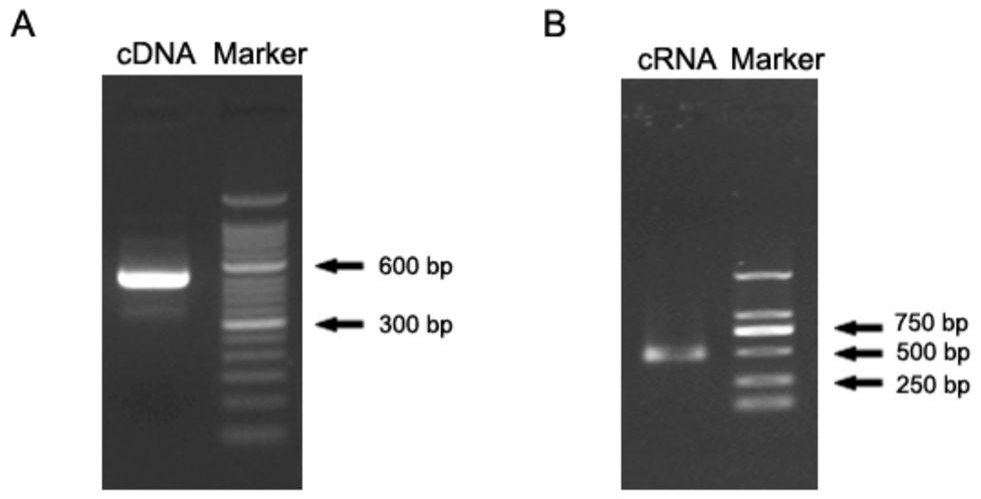
Agarose gel electrophoresis of HTNV cDNA and cRNA. (**A**) cDNA with a length of 516 bp was synthesized by reverse-transcription and subsequently amplified by PCR. Marker: 50 bp DNA ladder. (**B**) HTNV cRNA with a length of 499 bp was synthesized using the cDNA as template by *in* *vitro* transcription. Marker: DNA ladder DL2000.

Viral RNA was extracted from a HTNV virus stock, and cDNA was synthesized using viral RNA as the template and a combined, reverse transcription-polymerase chain reaction (RT-PCR). The length of the amplified cDNA products was 516 bp, which was the expected size (Figure 1). HTNV cRNA was synthesized *in vitro* using T7 RNA polymerase and the cDNA as templates. The size of the HTNV cRNA was 499 bp. The absorbance of HTNV cRNA was measured using a spectrophotometer, and the concentration was found to be 150.84 ng/μl. The copy number of the HTNV cRNA was thus calculated as 5.27×10^11^ copies/µl.

### Standardization of the SYBR Green Ⅰ-based qRT-PCR assay

**Figure 2:**
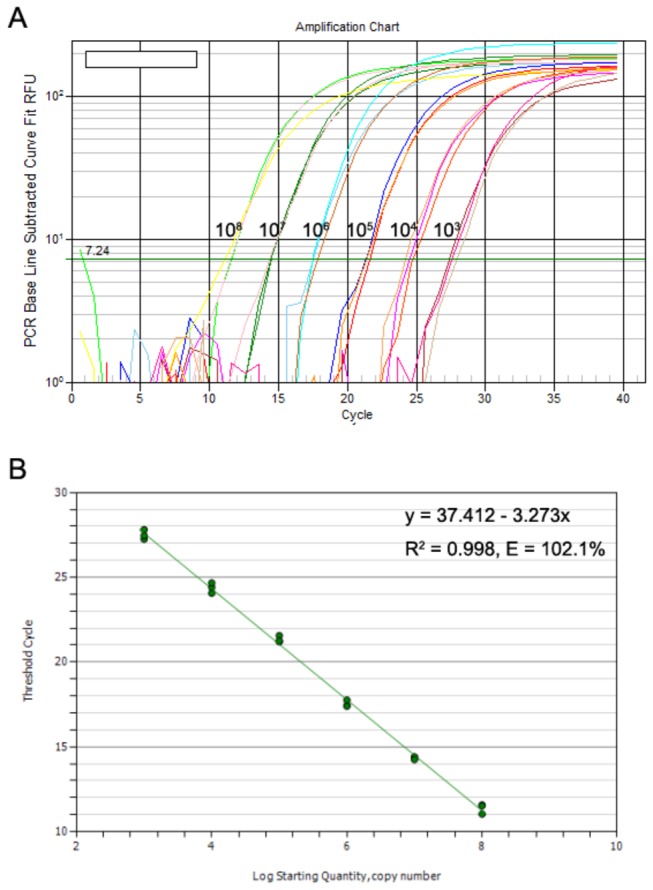
The HTNV cRNA amplification chart and standard curve. (**A**) 10-fold serial dilutions of the HTNV cRNA standards ranging from 1×10^8^ to 1×10^3^ copies/µl were amplified by qRT-PCR assay. (**B**) The HTNV cRNA standard curve was plotted by Ct values versus the HTNV cRNA log copy numbers in triplicate tests.

Using 10-fold serial dilutions of the synthesized HTNV cRNA templates, SYBR Green Ⅰ-based one-step qRT-PCR was performed to establish a standard curve. The external standard curve was generated by plotting the Ct values versus serial dilutions of the HTNV cRNA templates ranging from 1×10^8^ to 1×10^3^ copies/µl. The observed linearity was good for the standard curve over a wide range of HTNV cRNA dilutions in triplicate tests, and the correlation coefficient (R^2^) was 0.998. The slope of the curve was -3.273, and the amplification efficiency of the assay was 102.1% based on the slope of the exponential phase in the amplification chart. The cycle intervals of qRT-PCR between each dilution ranged from 2.9 to 3.8 cycles (Figure 2). 

### Optimization of the SYBR Green Ⅰ-based qRT-PCR assay

The annealing temperature of the assay was optimized at 57°C using a gradient of annealing temperatures from 51°C to 61°C. The optimal amount of input cRNA templates was 0.2 μg per reaction, and each primer in the reaction tube was used at a final concentration of 0.4 µM.

### Specificity of the SYBR Green Ⅰ-based one-step qRT-PCR

**Figure 3:**
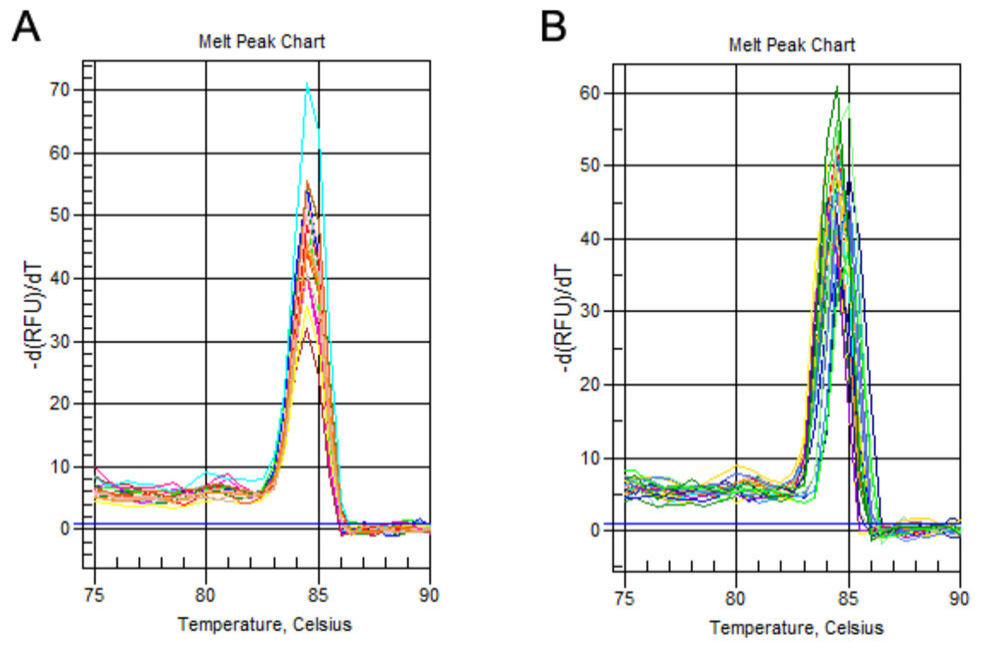
Melting peaks of the SYBR Green Ⅰ-based one-step qRT-PCR products. (**A**) Melting peaks of the qRT-PCR products obtained from *in* *vitro* transcribed HTNV cRNA ranging from 1×10^8^ to 1×10^3^ copies/µl. Only a single sharp peak at 84°C was visible in the melt peak chart. (**B**) Melting peaks obtained from serum samples of HFRS patients. All melting curves in A and B have similar shapes.

**Figure 4:**
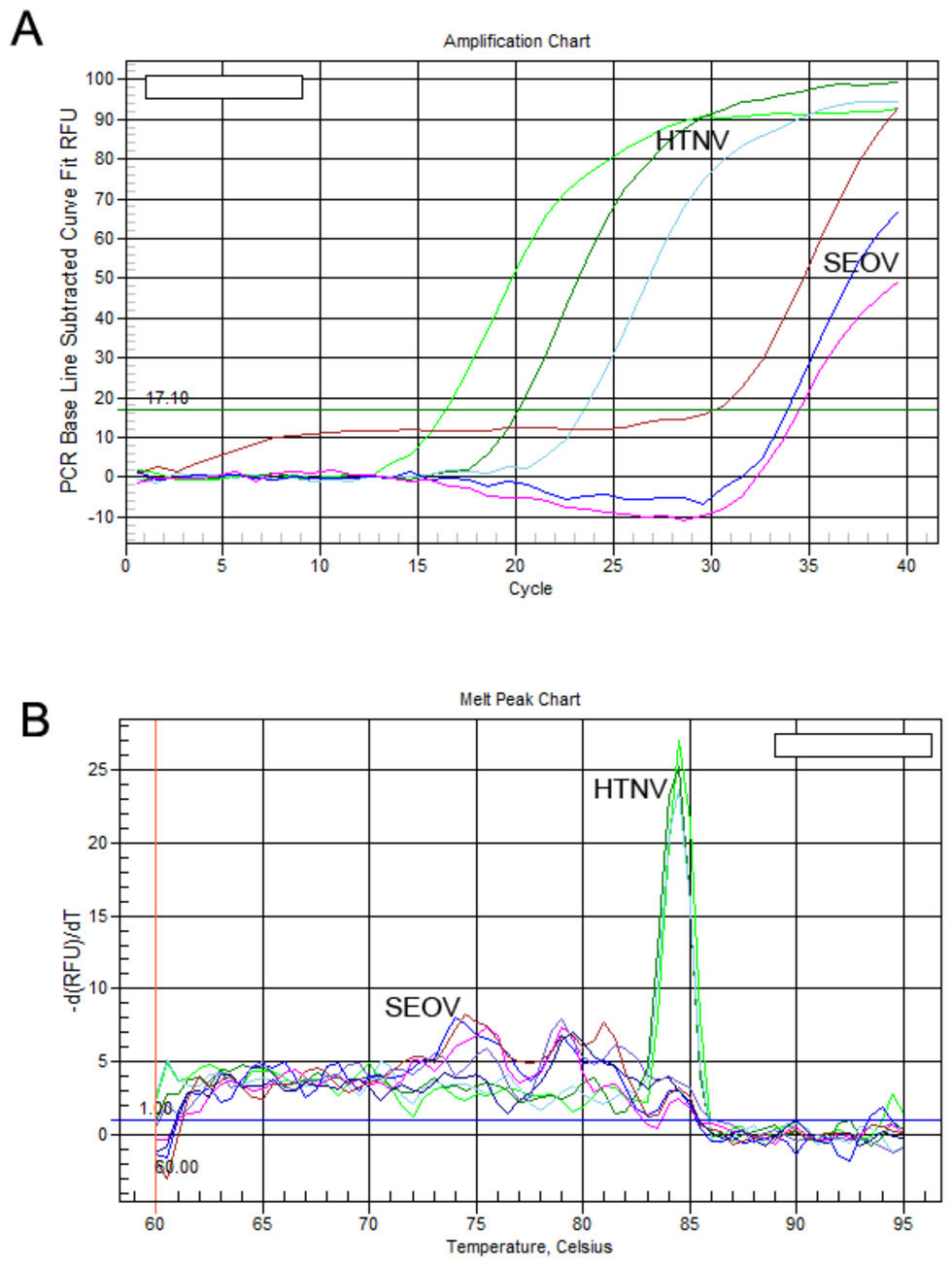
Amplification chart and melt peak chart of HTNV and SEOV. (**A**) Different dilutions of HTNV and SEOV RNA were tested using the SYBR Green Ⅰ-based one-step qRT-PCR assay, and only HTNV RNA was amplified. (**B**) A single sharp peak at 84°C in the melt peak chart indicated that only specific products of HTNV was obtained.

This one-step qRT-PCR assay was highly specific for detecting HTNV because RNA samples from SEOV (also in the genus *Hantavirus*) and other viruses (HBV, HCV, and HIV) were not amplified during the assay. Melting curve analysis on the qRT-PCR products showed that no primer-dimers or non-specific products were formed, and only a single, sharp peak at 84°C was visible in the melt peak chart for HTNV cRNA and serum samples from HFRS patients (Figure 3). Only HTNV was amplified when SEOV and HTNV were tested simultaneously using the assay, and no sharp peak was visible for the SEOV qRT-PCR products in the melting curve chart (Figure 4). 

### Sensitivity of the SYBR Green Ⅰ-based one-step qRT-PCR

**Figure 5:**
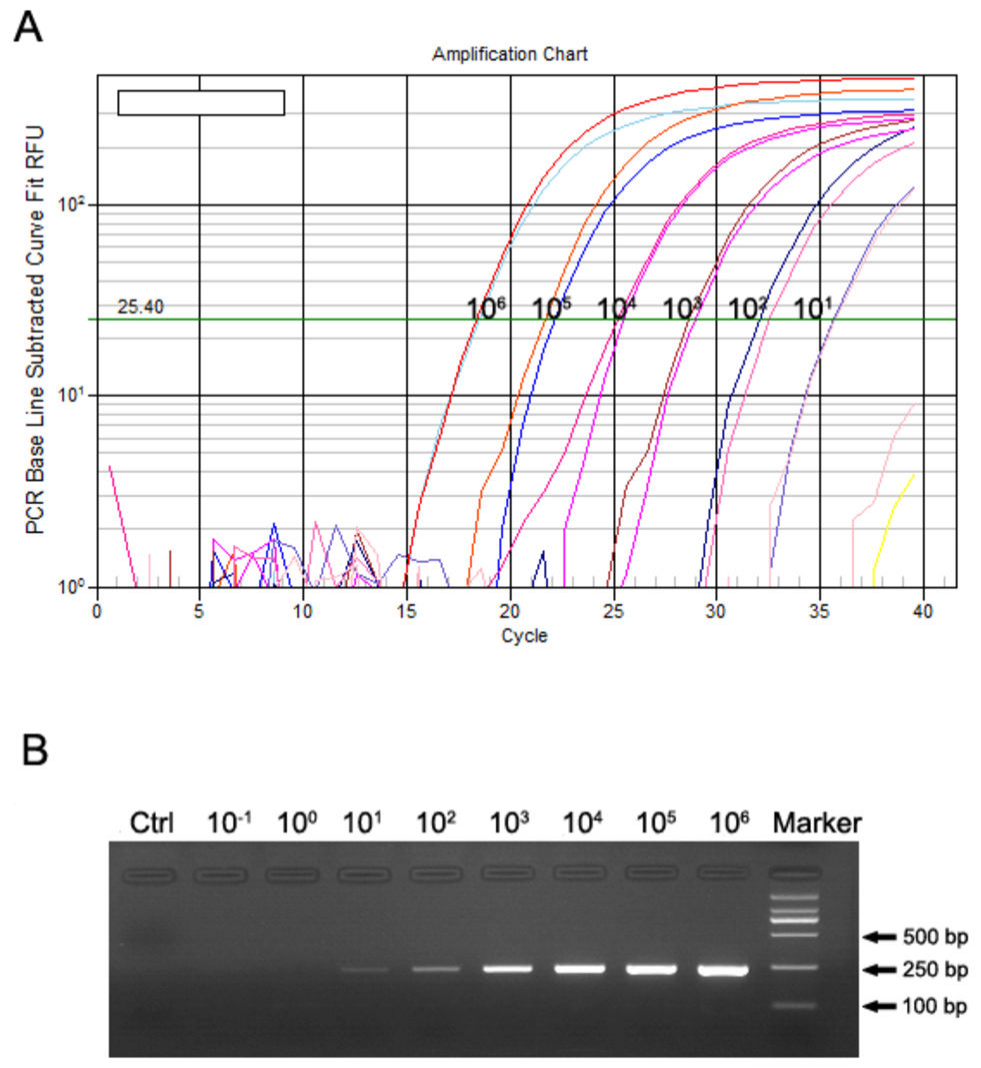
Sensitivity of the SYBR Green Ⅰ-based one-step qRT-PCR assay. (**A**) Serial dilutions of the HTNV cRNA standards ranging from 1×10^6^ to 1×10^-1^ copies/µl were amplified using the SYBR Green Ⅰ-based one-step qRT-PCR assay in duplicate, and the minimum detection limit of the qRT-PCR assay was 10 copies/µl. (**B**) The qRT-PCR products were electrophoresed on a 2% agarose gel. Marker: DNA ladder of DL2000.

The sensitivity of the qRT-PCR assay was evaluated by measuring 10-fold serial dilutions of the HTNV cRNA standards ranging from 1×10^6^ to 1×10^-1^ copies/µl in duplicate. The results indicated that the qRT-PCR assay could detect the HTNV RNA at levels as low as 10 copies/µl. The 237-bp amplicons of the qRT-PCR products were observed by electrophoresis on a 2% agarose gel (Figure 5).

### Intra- and inter-assay variations

The reproducibility of the qRT-PCR assay was established using the Ct values that were obtained from testing the HTNV cRNA standards in triplicate within each run (intra-assay) and in three different runs (inter-assay). The standard deviation (SD) and coefficient of variation (CV) values for the intra-assay ranged from 0.177 to 0.770 and from 0.68% to 3.00%, respectively. The SD and CV of the inter-assay were slightly higher than the intra-assay and ranged from 0.138 to 1.126 and from 0.86% to 3.21%, respectively, thus demonstrating good reproducibility (Table 1). 

**Table 1:** Reproducibility of the SYBR Green I-based one-step qRT-PCR in intra- and inter-assay.

**Assay**	**Copy numbers of the HTNV cRNA standards (copies/µl)**						
	1.00E+07	1.00E+06	1.00E+05	1.00E+04	1.00E+03	1.00E+02	1.00E+01
**intra-assay**							
Ct values (Mean±SD)	16.44±0.296	19.18±0.574	21.82±0.308	25.03±0.177	28.58±0.193	31.65±0.608	34.39±0.770
CV (%)	1.80%	3.00%	1.41%	0.71%	0.68%	1.92%	2.24%
**inter-assay**							
Ct values (Mean±SD)	16.06±0.138	18.22±0.337	21.90±0.215	25.48±0.379	29.15±0.703	32.25±0.280	35.05±1.126
CV (%)	0.86%	1.85%	0.98%	1.49%	2.41%	0.87%	3.21%

### Assay performance on clinical samples

To evaluate the usefulness of the assay for clinically detecting HTNV RNA, the protocol was applied to serum samples of 27 HFRS patients who had been diagnosed by clinical manifestations and laboratory examination. All 27 HFRS patients showed positive serum IgM and/or IgG test results for hantavirus antibodies. The peripheral blood were collected during the febrile phase of the illness (3-7 days after onset of fever), and the copy numbers of the HTNV RNA in the serum samples ranged from 2.06×10^1^ to 1.95×10^5^ copies/µl (Table 2). The copies of the HTNV RNA in the serum samples from HIV, HBV and HCV patients and normal controls were all below the level of detection. 

**Table 2:** Quantification of HTNV RNA in serum samples from patients and normal controls using the SYBR Green I-based one-step qRT-PCR assay.

**Sample Number**	**HTNV-IgM/IgG Antibodies Combo test**	**Days after onset of fever**	Severity of the HFRS disease *	**Copies/µl of the HTNV RNA**
HFRS 1	Positive/Positive	4	Critical	8.64×10^2^
HFRS 2	Positive/Positive	5	Critical	1.43×10^3^
HFRS 3	Positive/Positive	5	Severe	2.86×10^3^
HFRS 4	Positive/Positive	4	Moderate	4.23×10^2^
HFRS 5	Positive/Negative	6	Severe	1.49×10^4^
HFRS 6	Positive/Positive	3	Mild	1.47×10^4^
HFRS 7	Positive/Positive	6	Critical	1.83×10^4^
HFRS 8	Positive/Positive	6	Mild	1.25×10^3^
HFRS 9	Positive/Positive	4	Mild	5.52×10^2^
HFRS 10	Positive/Positive	2	Mild	1.05×10^5^
HFRS 11	Positive/Positive	4	Severe	1.95×10^5^
HFRS 12	Positive/Negative	6	Moderate	7.44×10^2^
HFRS 13	Positive/Negative	4	Moderate	1.78×10^3^
HFRS 14	Positive/Positive	4	Critical	1.23×10^4^
HFRS 15	Positive/Positive	4	Severe	1.06×10^4^
HFRS 16	Positive/Positive	4	Severe	3.64×10^4^
HFRS 17	Positive/Positive	5	Moderate	1.13×10^4^
HFRS 18	Positive/Positive	5	Mild	2.06×10^1^
HFRS 19	Positive/Positive	3	Moderate	3.26×10^1^
HFRS 20	Positive/Positive	3	Moderate	3.32×10^4^
HFRS 21	Positive/Negative	3	Mild	4.37×10^3^
HFRS 22	Positive/Positive	5	Moderate	8.39×10^3^
HFRS 23	Positive/Positive	3	Severe	1.62×10^2^
HFRS 24	Positive/Positive	4	Moderate	7.99×10^2^
HFRS 25	Positive/Negative	5	Mild	5.04×10^2^
HFRS 26	Positive/Positive	3	Mild	1.03×10^2^
HFRS 27	Positive/Negative	3	Moderate	1.63×10^2^
HBV 1	Negative/Negative	-	-	Negative
HBV 2	Negative/Negative	-	-	Negative
HBV 3	Negative/Negative	-	-	Negative
HCV 1	Negative/Negative	-	-	Negative
HCV 2	Negative/Negative	-	-	Negative
HCV 3	Negative/Negative	-	-	Negative
HIV 1	Negative/Negative	-	-	Negative
HIV 2	Negative/Negative	-	-	Negative
HIV 3	Negative/Negative	-	-	Negative
NC 1	Negative/Negative	-	-	Negative
NC 2	Negative/Negative	-	-	Negative
NC 3	Negative/Negative	-	-	Negative
NC 4	Negative/Negative	-	-	Negative

* , The severity of HFRS disease was classified on the basis of clinical manifestations and laboratory parameters used in the diagnostic criteria for HFRS in China as mild, moderate, severe, and critical. NC, Normal control.

## Discussion

The use of qRT-PCR in the field of virus research has increased greatly, and it has proved to be a useful tool for measuring the load of infectious virion in patient peripheral blood, especially for those infected with HIV, HBV or HCV. Changes in the viral load reflect the replication of the virus in the human body and indicate the severity of the disease, to some degree.

The major disadvantage of the SYBR Green Ⅰ-based PCR assay is that SYBR Green dye binds to any double-stranded DNA in the reaction, including primer-dimers and other non-specific amplification products, and could generate a false-positive signal, which would result in overestimation of the target concentration. However, with a well designed primer, SYBR Green can work well with spurious, non-specific background only showing up in the later cycles. In this study, we optimized the primer annealing temperature, component concentrations and reaction parameters to decrease the amount of primer-dimer and other non-specific reaction products. 

For quantitative PCR assays, the initial copy numbers of the targets can be calculated using the standard curve. In this study, a standard curve was established based on the serial dilutions of the HTNV cRNA standards. To construct the HTNV cRNA standards, the designed primer pair was modified with a T7-promoter sequence at the 5’-end of the forward primer and with an oligo-(dT) at the 5’-end of the reverse primer according to the method described by Totzke et al [19,20]. Incorporation of a T7-promoter sequence onto the forward primer was essential for performing *in vitro* transcription with the T7 RNA polymerase, and the addition of oligo-(dT) at the end of the reverse primer allowed for the generation of a cRNA sequence with a poly(A) tail at its 3’-end for stabilization. The use of two primer pairs ensured that the nucleotide sequences of the HTNV cRNA standards were identical to those in the HTNV genome. This method for the construction of cRNA standards was simple and is suitable to measure transcripts of any genes of interest. 

Recently, qRT-PCR assays based on the TaqMan probes have been developed for the detection of hantaviruses in rodents, cell culture or HFRS patients [16–18,21]. In this study, a SYBR Green Ⅰ-based one-step qRT-PCR assay coupled with melting curve analysis was established to quantify the HTNV RNA viral load. This method is highly specific and sensitive compared to conventional RT-PCR. It is also a high-throughput method for the detection and quantification of HTNV in clinical studies. The entire course of viral RNA extraction and qRT-PCR test consumed approximately three hours, making it a fast and simple assay. Due to the limitation of numbers of HFRS patients in the study, the clinical performance of the assay must be further evaluated. Additionally, the relationship between the HTNV RNA viral load and the severity of the disease must be analyzed, and the association of the dynamic changes in HTNV RNA copy numbers in the serum samples of HFRS patients with disease development must be monitored.

## Materials and Methods

### Cells and viruses

The HTNV strain 76-118 and SEOV strain R22 were used in this study. HTNV was passaged in Vero-E6 cells (American Type Culture Collection, CRL 1586) that were grown in MEM/EBSS medium (Thermo Fisher Scientific, Waltham, MA, USA) supplemented with 10% fetal bovine serum (FBS) (Life Technologies, Rockville, MD, USA). HTNV infection was monitored by indirect immunofluorescence assay (IFA) using mouse anti-NP mono-antibody and fluorescein isothiocyanate (FITC)-conjugated goat anti-mouse IgG antibody (SouthernBiotech, Birmingham, AL, USA). After 10 days of incubation, the cell monolayer was lysed by three freeze-thaw cycles and centrifuged at 4,000 g for 10 min. The supernatant was aliquoted and stored at -80 °C. 

### Viral RNA extraction

The RNA of hantaviruses (HTNV and SEOV) was extracted from collected virus stocks according to the instructions in the QIAamp Viral RNA Mini Handbook (Qiagen, Hilden, Germany). The viral RNA (60 µl) was eluted and stored at -80°C until use, and the concentration and quality of the eluted RNA were measured by UV absorbance at 260 nm and 280 nm using a Thermo Scientific NanoDrop^TM^ Spectrophotometer (NanoDrop Technologies, Wilmington, DE, USA).

### Clinical specimens and Ethics Statement

27 HFRS patients, 3 HBV patients, 3 HCV patients, 3 HIV patients and 4 normal controls were recruited from the Center of infectious diseases in the Tangdu Hospital from November, 2012 to January, 2013, and enrolled in the study. The diagnosis of HFRS was based on clinical manifestations and confirmed by the detection of hantavirus IgM/IgG antibodies using Hantavirus IgG/IgM Combo Test Card (Boson Biotech, Xiamen, China). Samples of peripheral blood from 27 HFRS patients were collected during the febrile phase of the illness (3-7 days after onset of fever) and stored at -80°C until use. HFRS disease severity was classified on the basis of clinical manifestations and laboratory parameters used in the diagnostic criteria for HFRS in China as mild, moderate, severe, and critical. The viral RNA extraction procedure was same as those described above. The clinical information about the HFRS patients, such as the days after onset of fever, the severity of the disease, was also collected and documented. The clinical study protocol was approved by the Ethics Committee of Fourth Military Medical University, and written informed consent was obtained from all enrolled subjects.

### Primer design and modification for the synthesis of HTNV cRNA

Alignments of HTNV S segment sequences encoding the NP of strain 76-118 (M14626.1) and other strains (A16 (AF288646.1), A9 (AF329390.1), and 84FLi (AY017064.1)) were performed based on the epidemiological data on HTNV epidemic strains in the Xi’an district, Shaanxi Province [4]. The primers 5’-ATCCTTTGTCGTCCCGATACTT-3’ (position of 417 to 438) and 5’-ATAGCCTTTGACTCCTTTGTCTCC-3’ (position of 864 to 887) were designed using Primer Premier 5.0 software (Premier Biosoft International, Palo Alto, CA, USA). The forward primer was modified by incorporating a T7-promoter sequence (5'-TAATACGACTCACTATAGGG-3') on its 5'-end of the primer. The reverse primer was modified by adding an oligo-(dT) to its 3'-end (File S1). The length of the constructed HTNV cRNA standards was 499 bp. 

### Amplification of cDNA template by conventional RT-PCR

The eluted HTNV RNA was reverse transcribed into cDNA using the RevertAid First Strand cDNA Synthesis Kit (Thermo Fisher Scientific, Waltham, MA, USA). The first-strand cDNA master mix (20 µl) was prepared as follows: 2 µl of template RNA was mixed with 1 µl of random primer (0.2 μg/μl), 4 µl of 5 × Reaction Buffer, 1 µl of RiboLock RNase Inhibitor (20 u/µl), 2 µl of 10 mM dNTP Mix, 1 µl of RevertAid M-MuLV Reverse Transcriptase (200 u/µl) and 9 µl of nuclease-free water. The reverse transcription reaction mixture was incubated for 5 min at 25°C followed by 60 min at 42°C.

The following PCR amplification was performed in 20 µl of mixture composed of 10 μl of DreamTaq^TM^ PCR Master Mix (2 ×) (Thermo Fisher Scientific, Waltham, MA, USA), 1 μl of each modified primer (10 μM), 2 μl of RT products and 6 μl of nuclease-free water. The PCR reaction was performed using a Bio-Rad MyCycler thermal cycler (Bio-Rad, Hercules, CA, USA) with the following parameters: 95°C at 2 min followed by 35 cycles of 30 s at 95°C, 30 s at 60°C and 30 s at 72°C and a final extension at 72°C for 7 min. After amplification, the PCR products were electrophoresed in a 2% agarose gel (Biowest, Madrid, Spain) and visualized using a Gel Doc System (Bio-Rad, Hercules, CA, USA).

### Precipitation of PCR products and *in vitro* transcription

The PCR products were precipitated with 3 M NaOAc and 100% ethanol and then washed once with 70% ethanol. Subsequently, the products were submitted to *in vitro* transcription using T7 RNA polymerase (TaKaRa Biotechnology, Dalian, China) to synthesize cRNA in accordance with the manufacturer’s instruction. HTNV cRNA was processed twice with DNase I (TaKaRa Biotechnology, Dalian, China) to remove residual DNA, and this step was followed by chloroform/isoamyl alcohol/ethanol precipitation. The HTNV cRNA transcripts were finally dissolved in 40 µl of RNase-free water, electrophoresed in a 2% agarose gel (Biowest, Madrid, Spain) and visualized by ethidium bromide. The HTNV cRNA stock solution was serially diluted and stored at -80°C until use. 

### Quantification of the HTNV cRNA

The concentration and quality of the HTNV cRNA was measured by UV absorbance at 260 nm and 280 nm in duplicate using a Thermo Scientific NanoDrop^TM^ Spectrophotometer (NanoDrop Technologies, Wilmington, DE, USA). The copy number of the HTNV cRNA standards was calculated using the following formula [22]: RNA copy number (copies/μl) = RNA concentration (g/μl) × 6.02 × 10^23^ (copies/mol)/345 × RNA length (b). 

### SYBR Green Ⅰ-based one-step qRT-PCR assay

qRT-PCR amplification was performed using a Bio-Rad iQ5 Multi-color Real-Time PCR Detection System (Bio-Rad, Hercules, CA, USA) with the One-Step SYBR PrimeScript RT-PCR Kit ii (TaKaRa Biotechnology, Dalian, China). Briefly, 2 µl of each HTNV cRNA standard was mixed with 12.5 µl of 2 × One-Step SYBR RT-PCR Buffer, 1 µl of PrimeScript One-Step Enzyme Mix 2, 1 µl of each primer (0.4 µM), and 7.5 µl of RNase-free water in 8-strip tubes (BIOplastics, Landgraaf, Netherlands). The forward primer, 5’-AAGCATGAAGGCAGAAGAGAT-3’ (position of 594 to 614), and the reverse primer, 5’-TAGTCCCTGTTTGTTGCAGG-3’ (position of 811 to 830), which amplified a 237 bp fragment, were designed using the Primer Premier 5.0 software (Premier Biosoft International, Palo Alto, CA, USA). The sequences of the primers were compared to sequence databases using the program BLAST on the NCBI website, and the primer pairs were synthesized and purified by Sangon (Sangon Biotech, Shanghai, China). The thermal cycling conditions comprised reverse transcription at 42°C for 5 min and 95°C for 10 s, followed by 40 cycles of PCR amplification at 95°C for 5 s and 57°C for 30 s. After amplification, a melting curve analysis program was used to verify the authenticity of the PCR products by their specific melting temperatures (Tm) in accordance with the manufacturer’s instructions. 

### Generation of a standard curve using the SYBR Green Ⅰ-based one-step qRT-PCR assay

To construct a standard curve, 10-fold serial dilutions of the HTNV cRNA standards spanning 1×10^8^ to 1×10^3^ copies/µl in Easy Dilution (TaKaRa Biotechnology, Dalian, China) were used and tested in triplicate. Standard curve was plotted as the mean Ct values versus the log HTNV cRNA copy numbers. Regression analysis, standard curve slopes and amplification efficiencies were calculated using automated software (Bio-Rad iQ5 2.0 Standard Edition Optical System Software). 

### Optimization of the SYBR Green Ⅰ-based one-step qRT-PCR assay

 Different parameters, such as the gradient of annealing temperature (ranging from 51 to 61°C), the gradient of primer concentrations and amounts of cRNA templates in 25 µl of reaction volume, were tested for the optimization of the amplification of specific HTNV products.

### Assay specificity

To investigate the specificity of the SYBR Green Ⅰ-based one-step qRT-PCR assay, SEOV and other viruses (HBV, HCV, and HIV) were subjected to qRT-PCR with the same amplifying primers and reaction conditions. 

### Assay sensitivity

The detection limit of the qRT-PCR was determined using 10-fold serial dilutions of the HTNV cRNA ranging from 1×10^6^ to 1×10^-1^ copies/µl in duplicate. 

### Assay reproducibility

Serial dilutions of the HTNV cRNA standards ranging from 1×10^7^ to 1×10^1^ copies/µl were assayed to evaluate the reproducibility, and three independent HTNV cRNA dilutions were tested in a single run to evaluate the intra-assay variance. The inter-assay variance was measured by testing each dilution in three independent runs, and the mean, SD and CV values were separately calculated for each HTNV cRNA dilution.

## Supporting Information

File S1 **Nucleotide sequences of the target S segment of the HTNV genome.** Underlined sequences were primers for the construction of the HTNV cRNA and the sequences in bold for the amplification of the target fragments with the SYBR Green I-based one-step qRT-PCR assay.
(DOC)
