# Correction: Quantification of Hantaan Virus with a SYBR Green Ⅰ-Based One-Step qRT-PCR Assay

**DOI:** 10.1371/annotation/59cb3bf3-cddd-470c-a94e-14992796f861

**Published:** 2013-12-30

**Authors:** Wei Jiang, Hai-tao Yu, Ke Zhao, Ye Zhang, Hong Du, Ping-zhong Wang, Xue-fan Bai

The third sentence under the section "SYBR Green I-based one-step qRT-PCR assay" in Materials and Methods is missing manufacturer information and a citation. The corrected sentence should read:

"The forward primer, 5’-AAGCATGAAGGCAGAAGAGAT-3’ (position of 594 to 614), and the reverse primer, 5’-TAGTCCCTGTTTGTTGCAGG-3’ (position of 811 to 830), which amplified a 237 bp fragment, were designed and synthesized by Sangon (Sangon Biotech, Shanghai, China) using the Primer Premier 5.0 software (Premier Biosoft International, Palo Alto, CA, USA) (Jiang, et al.)."

The full citation is:

Wei Jiang, Ping-zhong Wang, Hai-tao Yu, et al. (2013) Development of a SYBR Green I based one-step real-time PCR assay for the detection of Hantaan virus. J Virol Methods. doi: pii: S0166-0934(13)00474-6. 10.1016/j.jviromet.2013.11.004. PubMed: 24269331.

The legend of Figure 4A contains an omission. The correct legend should mention "cRNA". Please see the corrected legend here:

(A) Different dilutions of HTNV and SEOV RNA were tested using the SYBR Green Ⅰ-based one-step qRT-PCR assay, and only HTNV cRNA was amplified. (B) A single sharp peak at 84°C in the melt peak chart indicated that only specific products of HTNV was obtained. 

