# Supplementary material for: Quantification of Hantaan Virus with a SYBR Green Ⅰ-Based One-Step qRT-PCR Assay
Source: PLoS One. 2013 Nov 21;8(11):e81525. doi: 10.1371/journal.pone.0081525 (PMC3836762; doi:10.1371/journal.pone.0081525)
Supplement: File S1 — Nucleotide sequences of the target S segment of the HTNV genome. Underlined sequences were primers for the construction of the HTNV cRNA and the sequences in bold for the amplification of the target fragments with the SYBR Green I-based one-step qRT-PCR assay. (DOC) [file pone.0081525.s001.doc]

TAATACGACTCACTATAGGGATCCTTTGTCGTCCCGATACTTCTGAAAGCTCTGTATATGTTGACAACAAGGGGGAGGCAAACTACCAAGGATAATAAAGGGACCCGGATTCGATTTAAGGATGATAGCTCGTTCGAGGATGTTAACGGTATCCGGAAACCAAAACATCTTTACGTGTCCTTGCCAAATGCACAGTC**AAGCATGAAGGCAGAAGAGAT**TACACCTGGTAGATATAGAACAGCAGTCTGTGGGCTCTACCCTGCACAGATTAAGGCACGGCAGATGATCAGTCCAGTTATGAGTGTAATTGGTTTTCTAGCATTAGCAAAGGACTGGAGTGATCGTATCGAACAATGGTTAATTGAACCTTGCAAGCTTCTTCCAGATACAGCAGCAGTTAGCCTCCTTGGTGGT**CCTGCAACAAACAGGGACTA**CTTACGGCAGCGGCAAGTGGCATTAGGCAATATGGAGACAAAGGAGTCAAAGGCTATTTTTTTTTTTTTTTTTTTTTTTTTT
